# Crosstalk between N6-methyladenosine modification and circular RNAs: current understanding and future directions

**DOI:** 10.1186/s12943-021-01415-6

**Published:** 2021-09-24

**Authors:** Xin Wang, Rui Ma, Xilin Zhang, Lian Cui, Yangfeng Ding, Weimin Shi, Chunyuan Guo, Yuling Shi

**Affiliations:** 1 Department of Dermatology, Shanghai Skin Disease Hospital, Tongji University School of Medicine, 1278 Baode Road, Jing’an District, Shanghai, 200443 China; 2 Institute of Psoriasis, Tongji University School of Medicine, Shanghai, China; 3 Ministry of Education-Shanghai Key Laboratory of Children’s Environmental Health, Xinhua Hospital, Shanghai Jiao Tong University School of Medicine, Shanghai, China; 4 Department of Dermatology, Shanghai General Hospital, Shanghai Jiao Tong University School of Medicine, Shanghai, China

**Keywords:** N^6^-methyladenosine, Circular RNA, Crosstalk

## Abstract

N^6^-methyladenosine (m^6^A) is a prevalent internal modification in eukaryotic RNAs regulated by the so-called “writers”, “erasers”, and “readers”. m^6^A has been demonstrated to exert critical molecular functions in modulating RNA maturation, localization, translation and metabolism, thus playing an essential role in cellular, developmental, and disease processes. Circular RNAs (circRNAs) are a class of non-coding RNAs with covalently closed single-stranded structures generated by back-splicing. CircRNAs also participate in physiological and pathological processes through unique mechanisms. Despite their discovery several years ago, m^6^A and circRNAs has drawn increased research interest due to advances in molecular biology techniques these years. Recently, several scholars have investigated the crosstalk between m^6^A and circRNAs. In this review, we provide an overview of the current knowledge of m^6^A and circRNAs, as well as summarize the crosstalk between these molecules based on existing research. In addition, we present some suggestions for future research perspectives.

## Background

RNA modifications (e.g., N^6^-methyladenosine [m^6^A], 5-methylcytosine, pseudouridine, N^4^-acetylcytidine, ribose methylations, and N^1^-methylguanosine), have recently emerged as vital post-transcriptional epigenetic modulators of gene expression in eukaryotes [1, 2]. Among these RNA modifications, m^6^A represents the most common and well-studied to date. m^6^A is a reversible modification that methylated adenosine at the N^6^ position of almost every type of RNA molecule, including mRNAs, small nuclear RNAs, ribosomal RNAs, and non-coding RNAs [1–3]. m^6^A was first discovered in the 1970s and developed rapidly during the past few years due to the advances in high-throughput m^6^A sequencing and methylated RNA m^6^A immunoprecipitation [4]. Moreover, m^6^A has been demonstrated to exert critical molecular functions in modulating RNA maturation, localization, translation, and metabolism. m^6^A dynamically exists and is involved in a variety of physiological and pathological processes, including growth, development, aging and diseases [4–6].

Circular RNAs (circRNAs) are a class of endogenous RNAs with covalently closed single-stranded structures also present in eukaryotes [7, 8]. Most circRNAs are non-coding RNAs while a proportion of cytoplasmic circRNAs have the coding potential to be translated into peptides [9, 10]. These molecules were also discovered several years ago, but has recently attracted the attention of researchers due to the advances in high-throughput RNA sequencing and bioinformatics [11]. Similar to other types of RNAs, circRNAs are involved in the maintenance of the normal physiological function of the human body, as well as the occurrence and development of a variety of human diseases [11–13]. While distinct from other RNA molecules, circRNAs possess unique biogenesis, biology, and characterization. Therefore, they may present peculiarities in response to RNA modifications.

Recently, some scholars have combined these two recent hot topics to investigate the crosstalk between them. In this review, we provide an overview of the current knowledge of m^6^A as well as circRNAs, and summarize the crosstalk between m^6^A modification and circular RNAs based on existing research. In addition, we have found that many questions still remain unanswered in this area and present some suggestions for future research perspectives.

### RNA m^6^A modification

**Fig. 1:**
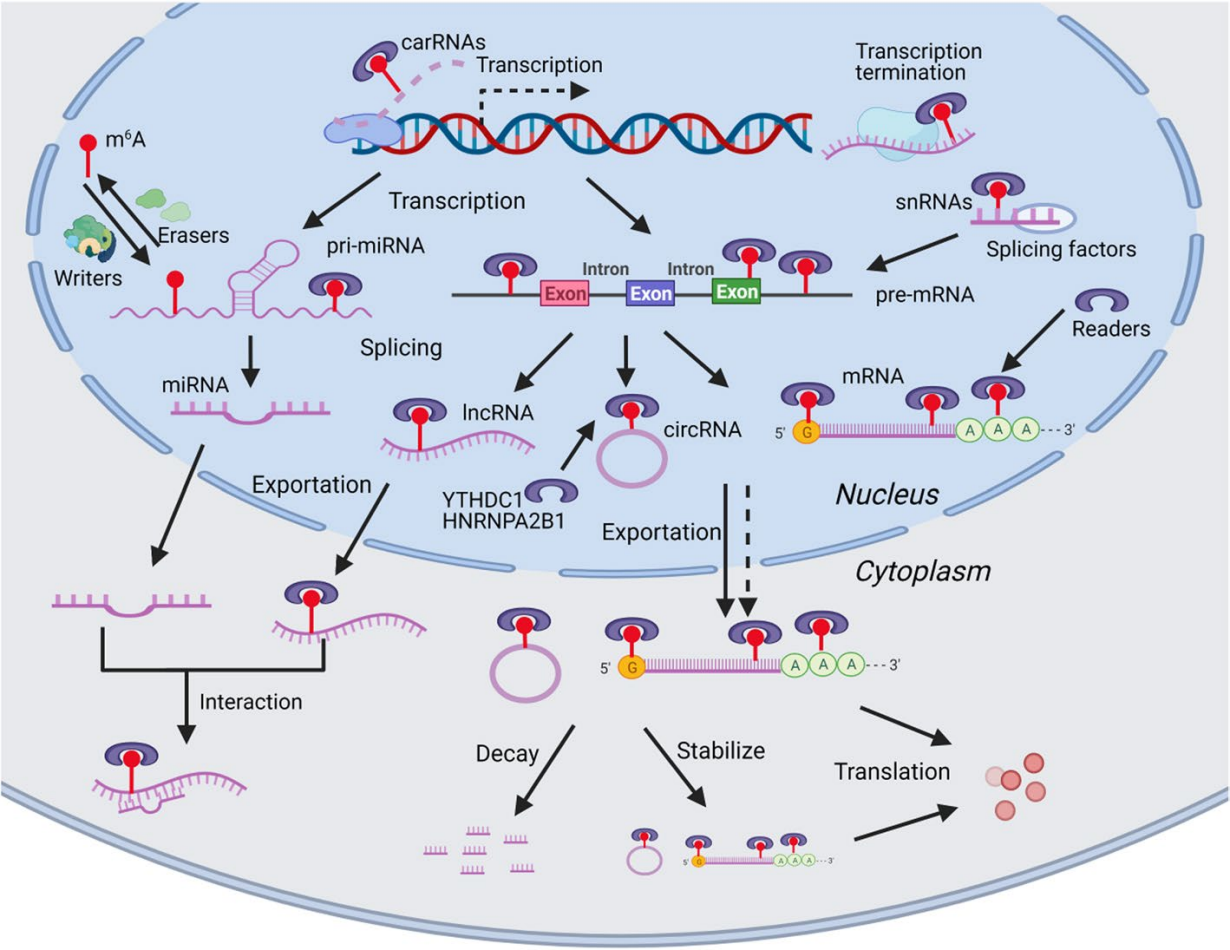
Overview of m6A modification. m^6^A modification is installed by the multicomponent m^6^A methyltransferases complex (writers) and removed by demethylases (erasers). The m^6^A modification is then identified by m^6^A readers which determine the fate of these RNAs and involved in various cellular processes. In the nucleus, m^6^A are identified by nuclear readers and modulates RNA transcription (transcription activation and termination), splicing (mRNAs, miRNAs, lncRNAs and circRNAs maturing) and structure (influence readers binding and splicing). Mature RNAs modified by m^6^A in the nucleus are recognized by readers, which subsequently mediate subcellular localization. In the cytoplasm, m^6^A are identified by cytoplasmic readers and modulates RNA stability (enhance stability or facilitate degradation), translation (promote translation via multiple mechanisms), and binding capacity (RNA-RBP interaction and RNA-RNA interaction)

Similar to DNA methylation, RNA m^6^A methylation is catalyzed and recognized by corresponding enzymes, methyltransferases- “writers”, demethylases- “erasers” and “readers”. Subsequently, these modified RNAs will present with a different fate in maturation, localization, translation and metabolism, thereby influencing various molecular cellular processes. The specific details are described below and a summary is presented in Fig. 1.

### Participants of m^6^A modification: writers, erasers, and readers

#### m^6^A writers

The m^6^A is installed by the multicomponent m^6^A methyltransferases complex (MTC), known as “writers”. The currently reported writers include methyltransferase-like 3 (METTL3), methyltransferase-like 14 (METTL14), methyltransferase-like 5 (METTL5), methyltransferase-like 16 (METTL16), Cbl proto-oncogene-like 1 (HAKAI), Wilms’ tumor 1-associating protein (WTAP), Vir Like M^6^A Methyltransferase Associated (VIRMA), RNA Binding Motif Protein 15/15B (RBM15/15B), Zinc Finger CCCH-Type Containing 4 (ZCCHC4), and Zinc Finger CCCH-Type Containing 13 (ZC3H13). These enzymes perform their respective duties and jointly complete the “writing” task. According to current knowledge, METTL3, METTL5, and METTL16 function as catalytic cores in the complex which catalyze m^6^A modification via methyltransferase domains [3–5, 14–16]. Other components typically play auxiliary roles, such as structural stabilization, reorganization of special RNA sites, and directing MTC location [3–5, 14–16].

#### m^6^A erasers

The m^6^A installed by writers can be removed by demethylases, or so-called “erasers”, which include fat mass and obesity-associated protein (FTO) and AlkB homolog 5 (ALKBH5) [14–16]. These two demethylases are localized in nuclear speckles and can oxidatively eliminate both DNA and RNA methylation, with a particularity for m^6^A methylation [15–17]. The identification of m^6^A demethylation provides evidence for the possible reversibility of the m^6^A modification.

#### m^6^A readers

To exert their biological functions, the m^6^A modifications determined by m^6^A writers and erasers must be identified by m^6^A readers. The currently reported readers include the YT521-B homology (YTH) domain family proteins (YTHDF1, YTHDF2, and YTHDF3), YTH domain containing proteins (YTHDC1 and YTHDC2), heterogeneous nuclear ribonucleoprotein (HNRNPC, HNRNPG, and HNRNPA2B1), insulin-like growth factor 2 mRNA-binding proteins (IGF2BP1, IGF2BP2, IGF2BP3), eukaryotic translation initiation factor 3 (EIF3), proline rich coiled-coil 2A (PRRC2A), and staphylococcal nuclease and tudor domain containing 1 (SND1). These RNA binding proteins (RBPs) have conserved m^6^A-binding domains that can specifically recognize m^6^A modifications. RBPs bind to m^6^A methylated RNAs and determine the fate of these RNAs, thus regulating various cellular processes such as transcription, splicing and maturing, exportation, translation, decay and others [3–5, 14–16]. Therefore, the m^6^A readers represent intermediaries for RNA m^6^A modification and different RNA fates.

A more comprehensive summary than previous reviews on the classification and functions of m^6^A writers, erasers, and readers is presented in Table 1. It appears that compared with a simple m^6^A installation and elimination function of writers and erasers, the roles of readers are more complicated and diverse, which is an area of keen research interest.

**Table 1:** Writers, erasers and readers of RNA m^6^A modification

Category	Factors	Roles	Refs
Writers	METTL3	m^6^A catalytic subunit	[14, 15]
	METTL14	Forms heterodimer with METTL3 to stabilize METTL3 and assist recognizing the subtract	[14, 18]
	METTL16	m^6^A catalytic subunit	[19]
	METTL5	Ribosome 18S m^6^A methyltransferase	[20, 21]
	TRMT112	Forms heterodimeric complex with METTL5 as a methyltransferase activator to stabilize METTL5	[21]
	ZCCHC4	Ribosome 28S m^6^A methyltransferase	[22, 23]
	HAKAI	Essential member of the MTC	[24]
	WTAP	Promotse m^6^A methyltransferase activity and localization in nuclear speckles	[25]
	VIRMA	Binds the MTC and recruit it to specific RNA region	[26]
	RBM15/15B	Binds the MTC and recruit it to specific RNA site	[27]
	ZC3H13	Promotes nuclear localization of MTC to modulate m^6^A in the nucleus	[28]
Erasers	FTO	Eliminates m^6^A by oxidation	[14–18]
	ALKBH5	Eliminates m^6^A by oxidation	[14–18]
Readers	YTHDF1	Facilitates the ribosome assembly of m^6^A-mRNAs and interacts with the initiation factor to promote translation; cooperates with YTHDF2 and YTHDF3 to mediate degradation of m^6^A-mRNAs	[29, 30]
	YTHDF2	Reduces m^6^A-mRNAs stability; stabilize m^6^A-mRNAs specifically in cancer stem cells; cooperates with YTHDF1 and YTHDF3 to mediate degradation of m^6^A-mRNAs	[29–31]
	YTHDF3	Cooperates with YTHDF1 and YTHDF2 to mediate degradation of m^6^A-mRNAs	[30, 32]
	YTHDC1	Promotes RNA splicing and translocation; facilitates the decay of m^6^A-modified chromosome-associated regulatory RNAs; together with its target m^6^A-RNAs to regulate chromatin modification and retrotransposon repression; regulate histone methylation	[33–38]
	YTHDC2	Facilitates the translation and decrease the abundance of m^6^A-RNAs; has 3′-5′ RNA helicase activity and decrease the stability of m^6^A-mRNAs	[39–41]
	HNRNPC/G	Responsible for pre-mRNA processing and affect the alternative splicing of target m^6^A-mRNAs	[42–44]
	HNRNPA2B1	Binds to m^6^A-containing pri-miRNAs to promote pri-miRNA processing; may regulate mRNA splicing by binding to m^6^A-containing pre-mRNAs; facilitates m^6^A modification and nucleocytoplasmic trafficking of mRNAs	[45, 46]
	IGF2BP1/2/3	Regulates m^6^A-RNAs stability, subcellular localization and translation	[47–49]
	EIF3	Facilitates translation of m^6^A-mRNAs by recruiting the 43S complex	[50, 51]
	PRRC2A	Enhances m^6^A-mRNAs stability	[52]
	SND1	Enhances m^6^A-RNAs stability	[53]

### Biological functions of m^6^A modification

The modulation of m^6^A methylation on RNAs begins during transcription and is largely dependent on the subcellular localization of writers, erasers, and readers. The writers are primarily localized in the nucleus, so the writing processes predominantly occur during the nuclear phase [14–18]. The eraser ALKBH5 mainly exists and functions as a demethylase in the nucleus, and the eraser FTO exerts demethylase activity both in nucleus and cytoplasm [14–18, 54]. Thus, the erasing processes may occur in the nucleus and cytoplasm. Some readers are localized and “read” m^6^A in the nucleus, which may influence nuclear processes, such as transcription and RNA splicing. In addition, some readers are able to assist with m^6^A-RNAs export from the nucleus to the cytoplasm. Readers in the cytoplasm may regulate cytosolic processes, such as translation and degradation.

#### m^6^A modulates RNA transcription, splicing, and structure

RNA m^6^A modification is a post-transcriptional regulation which appears not to be related to transcription; however, a recent study demonstrated that m^6^A modification on chromosome-associated regulatory RNAs (carRNAs), including promoter-associated RNAs, enhancer RNAs, and repeat RNAs, can induce carRNA decay by YTHDC1 and impact the open chromatin state and downstream transcription [36]. Moreover, RNA m^6^A modification play a critical role in transcription termination by facilitating the formation of co-transcriptional R-loops to decrease the readthrough activity of Pol II [55]. Reports have confirmed that m^6^A modification on primary miRNAs (pri-miRNAs) promotes the recognition and processing by the microRNA microprocessor complex protein, DGCR8, thereby enhancing miRNA maturation [45, 56]. The regulation of m^6^A on pre-mRNA splicing has been validated in *Drosophila* [57], whereas the precise regulation pattern remains largely unknown in mammals. Nevertheless, some efforts have been made to consummate the pathways through which m^6^A modulates pre-mRNA splicing in mammals. For example, the m^6^A reader, HNRNPG, may use Arg-Gly-Gly motifs to co-transcriptionally interact with RNA polymerase II and m^6^A-modified nascent pre-mRNA to modulate alternative splicing [44]. Additionally, YTHDC1 can recruit and promote pre-mRNA splicing factors to enter the binding regions of targeted mRNAs to modulate mRNA splicing [33]. In addition, the m^6^A modification on U2 and U6 snRNAs may influence the splicing of specific pre-mRNA transcripts [58, 59]. Evidence also shows that m^6^A can alter RNA structures to affect RNA-protein interactions in cells. For instance, m^6^A alters the local structure in mRNA and lncRNA and thereby influences the binding of HNRNPC to mediate pre-mRNA processing [42]. m^6^A located near splice sites in nascent pre-mRNA modulates HNRNPG binding, which influences RNAPII occupancy patterns and promotes HNRNPG-mediated alternative splicing [43, 44].

#### m^6^A modulates RNA subcellular localization

Mature RNAs modified by m^6^A in the nucleus are recognized by readers, which subsequently mediate subcellular localization. In general, nuclear readers (e.g., YTHDC1 and HNRNPA2B1) can identify m^6^A-RNAs (e.g., mRNAs and circRNAs), and accelerate their exportation from the nucleus to the cytoplasm [34, 45, 60]. However, for some RNAs, m^6^A modification may detain them within the nucleus. For example, m^6^A modification of lncRNA *RP11* can increase its accumulation in the nucleus and on chromatin, which may be due to its interaction with HNRNPA2B1 [61]. Interestingly, several RNAs without m^6^A modification can still be exported from the nucleus, indicating that the m^6^A is a facilitator but not an indispensable factor for translocation [14].

#### m^6^A modulates RNA stability, translation, and binding capacity

RNA exported to the cytoplasm may exert their biological functions or be degraded, and m^6^A modification can impact these processes via multiple cytoplasmic readers. Readers mediate the degradation of m^6^A-mRNAs, including YTHDF1, YTHDF2, YTHDF3, and YTHDC2, and readers enhance m^6^A-mRNA stability, including IGF2BPs, PRRC2A, and SND1 (Table 1) [29–32, 39–41, 47, 52, 53]. Moreover, these readers may regulate RNA stability through diverse mechanisms. For example, the carboxy-terminal domain of YTHDF2 selectively interacts with m^6^A-mRNAs, whereas the amino-terminal domain mediates the transposition of the YTHDF2-mRNA complex from the translatable pool to mRNA decay sites (e.g., processing bodies) [62]. IGF2BPs probably recruit RNA stabilizers, such as ELAV like RNA binding protein 1 (ELAVL1 or HuR), matrin 3 (MATR3), and poly(A) binding protein cytoplasmic 1 (PABPC1), to maintain the stability of their target m^6^A-RNAs [47]. Numerous studies have demonstrated that m^6^A can regulate translation with the assistance of readers, including YTHDF1, YTHDF2, YTHDF3, YTHDC2, IGF2BPs, and EIF3 [29–32, 39–41, 47–49], which involves several distinct mechanisms. For example, YTHDF1 can facilitate the ribosome assembly of m^6^A-mRNAs and interact with the initiation factor to promote translation [29]. In the absence of the cap-binding factor eIF4E, EIF3 can directly bind to the m^6^A in the 5′ untranslated region (UTR) and recruit the 43S complex to initiate translation [50]. METTL3 directly binds to the eukaryotic translation initiation factor 3 subunit h (eIF3h) and presumably promotes translation through ribosome recycling [63]. Promoter-bound METTL3 induces m^6^A in the coding region of mRNA to enhance translation by relieving ribosome stalling [64]. Moreover, m^6^A on 18S and 28S ribosomal RNA also play critical roles in the maintenance of ribosomal translation dynamics [20, 22]. Apart from the influence of the RNA-RBP interaction described above, m^6^A may also be indispensable for some RNA-RNA interactions. For example, the sufficient enrichment of the m^6^A modification on *linc1281* is required for the interaction between *linc1281* with miRNAs [65], and the m^6^A modified 353–357 region in the *YAP* 3’UTR was found to be critical for miR-582-3p targeting [66].

### CircRNAs

**Fig. 2:**
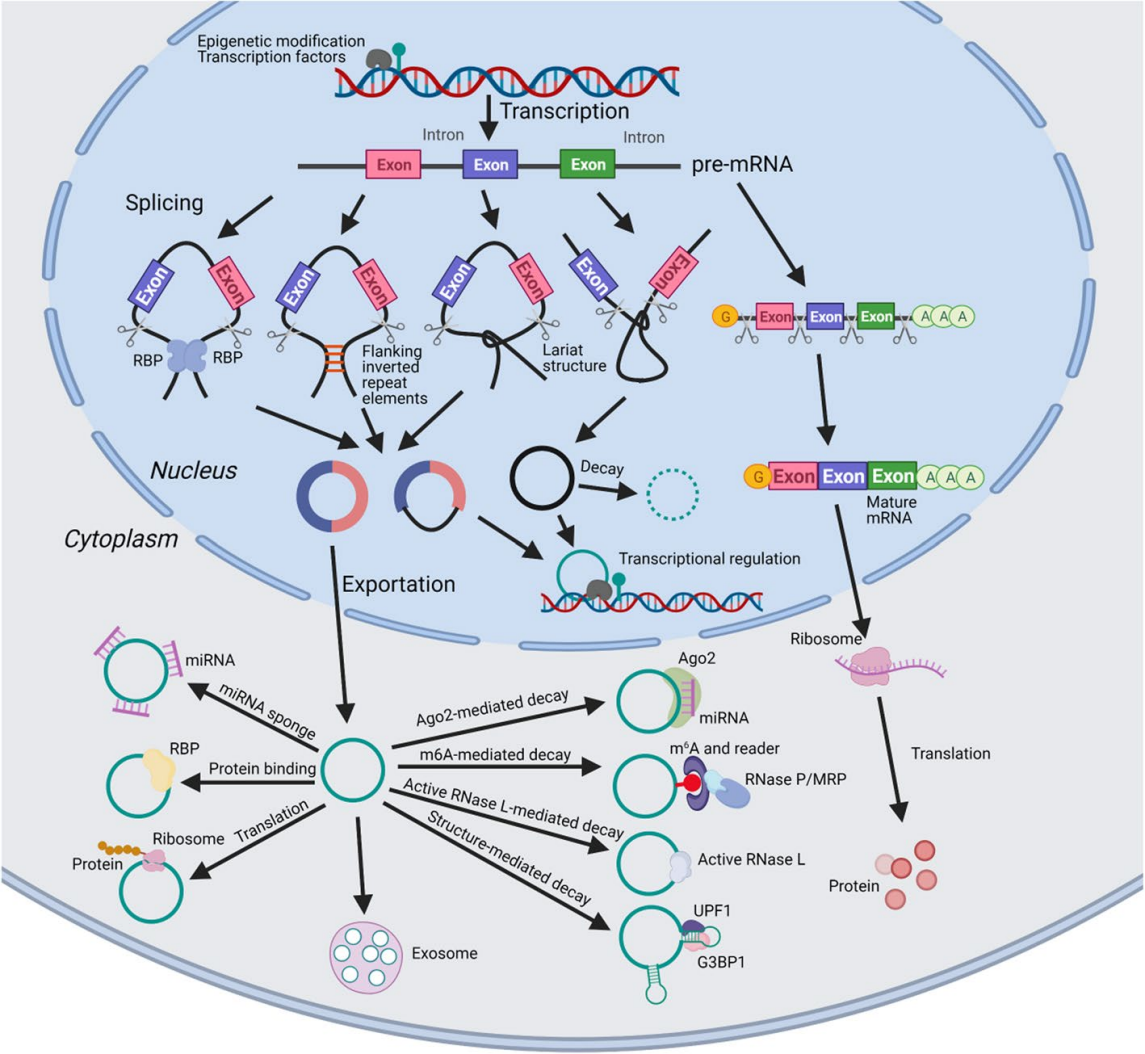
Summary of circRNA biology. Both circRNAs and mRNAs are originated from pre-mRNAs transcribed from genomic DNA which are regulated by transcriptional regulators. Distinguished from mRNA maturation (5′-capping, 3′-polyadenylation and introns removing), circRNA maturation go through various back-splicing processes which competes with the traditional mRNA splicing. CircRNAs matured in the nucleus may stay in the nucleus (EIcRNAs and ciRNAs with intron elements) or export to the cytoplasm (EcRNAs without introns). CircRNAs stay in the nucleus may participate in nuclear processes such as transcriptional regulation. CircRNAs export to the cytoplasm are involved in cytoplasmic processes such as miRNA sponging, RBP binding and translation. Some circRNAs are also enriched and stable in exosomes and secreted to extracellular components. All the circRNAs will eventually be degraded via a variety of mechanisms

In contrast to other RNA molecules, circRNAs have a unique circular structure, which requires a unique biogenesis process. Moreover, this stable structure may endow them with distinctive cellular functions, as well as unique approach to degradation. The associated details are described below and a summary is presented in Fig. 2.

### Biogenesis of circRNAs

Similar to mRNA maturation, the biogenesis of circRNAs should also undergo processes, including the transcription and splicing of pre-mRNAs [7, 8, 11]. Therefore, the factors that regulate transcription (e.g., epigenetic modifications and transcription factors) may influence the generation of circRNAs [7, 8, 11]. Distinguished from the canonical splicing of mRNAs, the alternative splicing of circRNAs represents a unique mode that competes with mRNA splicing. Instead of the 5′-capping, 3′-polyadenylation and introns removing events of mRNA maturation, a downstream 5′-splice site and a 3′-splice site are connected to form a covalently closed single-stranded structure in circRNA splicing [7, 8, 11–13]. There are several acknowledged mechanisms that can be used to interpret the circularization of circRNAs [7, 13, 67]. The first is intron pairing-driven circularization, in which the flanking of inverted repeat elements form RNA double strands through base-pairing. Another model is RBP-mediated circularization, in which the RBPs bind to the upstream and downstream flanking introns to form dimers. The third is lariat-driven circularization, which is mediated by the lariat structures that form in the exon-skipping events during linear splicing or intronic lariats that escape from the debranching of canonical linear splicing. These regulatory modes serve to bring the downstream splice-donor sites into close proximity with the upstream splice-acceptor site subject to alternative splicing. Based on the involved splicing and the genomic elements, three types of circRNAs are generated: 1) exonic circRNAs (EcRNAs), which are composed by one or more exons; 2) exon-intron circRNAs (EIcRNAs) that contain both exon and intron components; and 3) intronic circRNAs (ciRNAs), which consist of only introns [11, 68, 69].

### Exportation and distribution of circRNAs

As described above, the biogenesis of circRNAs occurs in the nucleus, and are then exported to the cytoplasm or detained in the nucleus. In general, most circRNAs are exported into the cytoplasm and the vast majority of cytoplasmic circRNAs are EcRNAs without introns [7, 12, 13]. Similar to many linear RNAs, circRNAs involving intron elements (e.g., EIcRNAs and ciRNAs) are usually sequestered in the nucleus [7, 12, 13]. There are some underlying mechanisms may interpret the exportation and distribution of circRNAs. The first and the most recently concerned is m^6^A-mediated circRNA translocation, which will be discussed in detail below. Another mechanism is the length-dependent evolutionarily conserved pathway which involves the association of circRNA lengths with the conserved proteins, UAP56 and URH49 [70]. In addition, since circRNAs are also enriched and stable in exosomes, they also widely exist in extracellular components [71, 72].

### Biological functions of circRNAs

Based on the unique characteristics and distribution, circRNAs may exert various biological functions. CircRNAs enriched in the nucleus are more likely to modulate transcription and splicing and several underlying mechanisms have been reported. For instance, *circSCMH1* may interact with transcription factor methyl CpG binding protein 2 (MeCP2) to restrain its transcriptional activity [73]. *circMRPS35* can recruit the histone acetyltransferase, KAT7, to elicit the acetylation of H4K5 in the promoters and directly bind to the promoters of *FOXO1* and *FOXO3a* genes to activate the transcription [74]. CircRNAs derived from exon 6 of the *SEP3* gene in Arabidopsis can bind to its cognate DNA locus to form an RNA:DNA hybrid, pausing transcription and exon 6 skipping in the alternative splicing of *SEP3* pre-mRNA [75].

Compared with nuclear circRNAs, cytoplasmic circRNAs are better acquainted. The most frequently reported function of circRNAs is their capacity to act as miRNA sponges. Such sponging refers to the manner by which circRNAs impair miRNA activity through sequestration in a competing endogenous RNA (ceRNA) manner, thereby raising the expression of miRNA target genes [76, 77]. Compared with this explicit inhibitory role on miRNA, circRNAs exhibit diverse binding effects on various proteins [78–81]. For example, circRNAs may not only recruit RBPs to stabilize and translate mRNAs, but also competitively bind to these RBPs to inhibit translation and degradation [62, 82, 83]. In addition, the interaction between RBPs and circRNAs may also influence the functionality and induce degradation through the ubiquitination of RBPs [84, 85].

CircRNAs are once considered as non-coding RNAs, however, recent studies have demonstrated that some cytoplasmic circRNAs carrying an initiation codon and putative open reading frames can be translated into peptides. Although lacking the traditional initiation elements (e.g., 5′ and 3’untranslated regions), circRNAs carrying an internal ribosome entry site (IRES) may undergo translation in a cap-independent manner [9, 86]. In addition, some circRNAs possess m^6^A and translation initiation sites may also go through m^6^A-driven translation with the assistance of the initiation factor, eIF4G2, and m^6^A reader, YTHDF3 [87]. Due to the same ORF components, several peptides translated by circRNAs are closely related to the proteins translated by their corresponding mRNAs. These peptides may act as substitutes to protect intact proteins from degradation or function as competitors to compete for regulators with intact proteins [88–90]. As proteins, while they may also play other functions [91–93], there are few relevant published studies, and further explorations remain to be performed.

Based on abundance and stability, circRNAs located in exosomes can be detected in the circulation and urine. Accumulating studies have confirmed that the circRNA content in the exosomes of some diseases is anomalous, indicating that they are promising diagnostic molecular markers [71, 72]. It is also feasible that cells transfer circRNAs to other cells or even throughout the body via excretion in exosomes. Therefore, they may act as mediators to ensure natural cell-to-cell communications. Besides, exosomes may bring abnormal amount of circRNAs to target cells, which is also an important source of various pathophysiological processes [71, 72].

### Degradation of circRNAs

Although the structure is highly stable and are resistant to exonucleases [7, 8, 11], they will eventually be degraded through the involvement of several unique and diverse degradation pathways. The binding of miRNAs to circRNAs can initiate the Argonaute 2 (Ago2)-mediated RNA decay, which is executed by the RNA-induced silencing complex (RISC) [94]. However, this phenomenon may not be as common as expected since similar to linear RNAs, the overwhelming majority of circRNAs bear sequences that are only partially complementary to miRNAs [13]. CircRNAs modified by m^6^A may be decayed by the ribonuclease complex RNase P/MRP, which will be discussed in detail below. In addition, there is a structure-mediated RNA decay model (e.g., high overall 3′ UTR structure) formed by base pairing in circRNAs that can be targeted and degraded by UPF1 and G3BP1 [95]. Upon viral infection, circRNAs can also be globally degraded by activated RNase L, which is required for PKR activation [96]. Actually, the above-mentioned degradation pathways are also suitable for some other RNA molecules and not unique to circRNAs.

### Crosstalk between m^6^A and circular RNAs

**Fig. 3:**
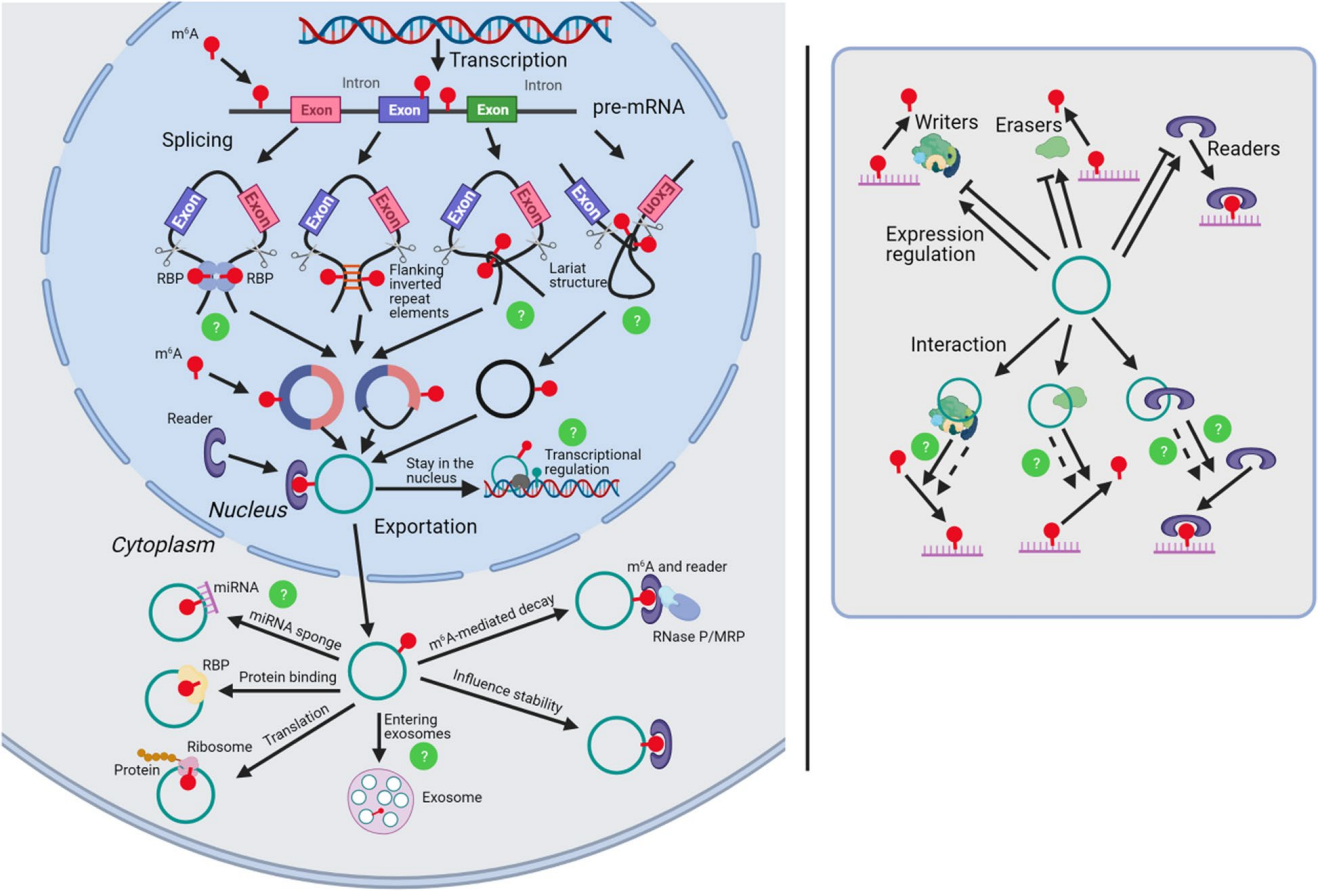
Crosstalk of m6A with circular RNAs. m^6^A modification is involved in the life cycle of circRNAs. First, m^6^A modification on pre-mRNAs may influence the splicing and generation of circRNAs. Second, m^6^A modification on mature circRNAs could affect the nuclear exportation of circRNAs. Third, m^6^A modification on circRNAs may influence the molecular functions of circRNAs, including transcription regulation, miRNA sponging, RBP binding and translation. Fourth, m^6^A modification may influence circRNA degradation or even entering the exosomes. Also, circRNAs can regulate m^6^A by affecting the expression or the functions of the m^6^A writers, erasers and readers. There are some predicted interactions between m^6^A and circRNAs have not been validated yet, which are marked with “?” in the figure

Through the above summary, we can find that there are many intersections between the regulatory pathway of m^6^A and the life cycle of circRNAs. Indeed, there have been many studies focusing on the crosstalk between m^6^A and circRNAs. Details are described below and a summary is shown in Table 2 and Fig. 3.

**Table 2:** Crosstalk between m^6^A and circRNAs

Crosstalk	circRNA	Roles	Refs
m^6^A regulates circRNAs expression	circMETTL3	METTL3 facilitates circMETTL3 expression in an m^6^A-dependent manner	[97]
	circ1662	METTL3 induced circ1662 generation by binding its flanking sequences and installing m^6^A modifications	[98]
	circCUX1	METTL3 mediates the m^6^A methylation of circCUX1 and stabilizes circCUX1	[99]
	circRNA-SORE	m^6^A modification raises circRNA-SORE level by increasing RNA stability	[100]
	circRNAs	m^6^A modification cause circRNAs selectively degraded by RNase P/MRP complex	[101]
m^6^A regulates circRNAs distribution	circGFRα1	METTL14 promotes cytoplasmic export of m^6^A-modified circGFRα1 through the GGACU motif	[102]
	circNSUN2	m^6^A modification of circNSUN2 facilitates cytoplasmic export	[60]
m^6^A regulates circRNAs function	circRNAs	Extensive m^6^A modifications in circRNAs drives protein translation in a cap-independent fashion	[87]
	circRNAs	m^6^A modification controls circRNA immunity	[103]
circRNAs regulate m^6^A	hsa_circ_0072309	hsa_circ_0072309 upregulates the expression of m^6^A demethylase FTO by targeting miR-607	[104]
	circMAP2K4	circMAP2K4 promote YTHDF1 expression by binding with hsa-miR-139-5p	[105]
	circRAB11FIP1	circRAB11FIP1 regulated the m^6^A methylation of ATG5 and ATG7 mRNA via upregulating FTO	[106]
	circMEG3	circMEG3 inhibits the expression of METTL3 dependent on HULC	[107]
	circNOTCH1	circNOTCH1 regulates the m^6^A modification on Nothch1 mRNA by binding to METTL14.	[108]
	circZbtb20	circZbtb20 enhances the interaction of ALKBH5 with Nr4a1 mRNA, leading to ablation of the m^6^A on Nr4a1 mRNA	[109]
	circSTAG1	circSTAG1 regulates m^6^A modification on FAAH by mediating ALKBH5 translocation	[110]

### m^6^A modulates the expression of circRNAs

Similar to other RNA molecules, m^6^A can also modulate the expression of circRNAs through regulating their generation, stability, or degradation. A recent study confirmed that METTL3 can install m^6^A in the reverse complementary sequences of flanking introns of *circ1662*, as well as facilitate the generation of *circ1662* based on the intron pairing-driven circularization pattern [98]. Moreover, circRNAs modified by m^6^A can be recognized by readers and exhibit changes in stability, resulting in altered expression [99, 100]. A subset of m^6^A-containing circRNAs may be endoribonucleolyticly degraded by the RNase P/MRP complex, which depends on the cooperative binding of HRSP12 and YTHDF2 [101]. The dynamic balance or the imbalance of circRNA expression is a consequence of the combined effect of these regulatory factors.

### m^6^A modulates the distribution of circRNAs

Some studies have demonstrated that the m^6^A modification on circRNAs may modulate their nuclear exportation [60, 102]. This process may depend on the recognition and mediation of m^6^A readers [60]. Despite these initial findings, the mechanisms underlying the subcellular trafficking of m^6^A-circRNAs remains largely unknown.

### m^6^A modulates the function of circRNAs

As described above, although considered to be non-coding RNAs, some circRNAs have the potential to encode proteins. Due to their unique structure, the translation of m^6^A modified circRNAs also differs from that of the linear RNAs. In this process, m^6^A-circRNAs are identified by the reader, YTHDF3, which recruits the translation initiation factors, eIF4G2 and eIF3A, to initiate translation in a cap-independent manner [87]. The m^6^A modification also influences the function of circRNAs in the regulation of innate immunity. Unmodified foreign circRNAs can directly trigger RIG-I signaling to promote immune activation; however, m^6^A-circRNAs may recruit YTHDF2 to form a complex with RIG-I and suppress the RIG-I immune signaling [103].

### circRNAs modulates m^6^A

Conversely, circRNAs can also regulate m^6^A modification. Some circRNAs can regulate m^6^A by affecting the expression of the m^6^A writers, erasers, and readers [104–107]. Other circRNAs may influence the functions of m^6^A writers, erasers, and readers [108–110]. For example, circRNAs can competitively bind to writers and contend modifications, thereby hindering the modification of other RNAs by writers [108]. In addition, circRNAs can not only recruit ALKBH5 to ablate the m^6^A modification of target mRNA, but also capture ALKBH5 to suppress its translocation into the nucleus and impede its role of ablating m^6^A [109, 110]. Generally, circRNAs can only mediately modulate m6A modification by regulating m6A writers, erasers or readers instead of directly affect m6A modification by themselves.

## Conclusions and prospectives

m^6^A modification and circRNA biology are undoubtedly current research hotspots, and the crosstalk between the two has attracted increasing attention from the researchers. In this review, we describe the complexity of m^6^A modification and circRNA biology, and present the identified crosstalk between them. Although some efforts have been devoted in this field, the study of correlation between m^6^A and circRNAs remains in the initial stages. In consideration of the current research realities, lots of questions remain to be addressed. In reference to the crosstalk mentioned in the previous section, we will present some perspectives which may represent potential future hot topics. These perspectives are also displayed in Fig. 3.

First, previous studies have only reported that m^6^A in the reverse complementary sequences of flanking introns facilitates the generation of circRNA through the intron pairing-driven circularization pattern [98]. However, other studies have verified that the m^6^A modification in pre-mRNA may regulate RNA-protein interactions and pre-mRNA processing [42–44]. This regulatory mode has been investigated in mRNA maturation but not in circRNAs biogenesis. Here, we propose that the m^6^A modification in pre-mRNA may affect the binding of some RBPs and regulate the generation of circRNA via RBP-mediated circularization. Whether m^6^A modification can modulate the lariat-driven circularization of circRNAs is also a topic worth examining.

Second, although it has been confirmed that m^6^A can regulate the stability of circRNAs [99, 100], in consideration of the specific circular structure and degradation pathway of circRNAs, is there any difference between circRNAs and linear RNAs in the mechanism of m^6^A regulating stability? Similarly, does the regulation of m^6^A on circRNAs exportation differ from its regulation on linear RNAs? In addition, studies have revealed that m^6^A may modulate the degradation of circRNAs [101]; however, whether this mode can participate in the normal life process or disease development has not yet been explored.

Third, while m^6^A are important initiators in the translation of circRNAs [88], it is unknown whether they are translation sustainers or terminators. Since m^6^A is able to affect RNA-RBP interactions [47], it may also influence the binding of RBPs to circRNAs. Similarly, the m^6^A modification on lncRNA is important for the binding of miRNAs [66], and there is an excellent probability that the m^6^A modification on circRNAs affects miRNA binding. Due to the recent research focus on miRNA sponging, potential m^6^A-mediated miRNA-circRNA interactions may represent another area of research interest.

Fourth, circRNAs can contend the modification of m^6^A writers [108], which may also apply to erasers and readers. In addition, while CircRNAs can recruit m^6^A erasers to ablate m^6^A modifications, it remains unknown whether they can also recruit m^6^A writers and readers to install and identify the m^6^A modifications.

## Data Availability

Not applicable.

## Abbreviations

m6A: N6-methyladenosine
circRNAs: Circular RNAs
MTC: Methyltransferases complex
METTL3: Methyltransferase-like 3
METTL14: Methyltransferase-like 14
METTL5: Methyltransferase-like 5
METTL16: Methyltransferase-like 16
HAKAI: Cbl proto-oncogene-like 1
WTAP: Wilms’ tumor 1-associating protein
VIRMA: Vir Like M6A Methyltransferase Associated
RBM15/15B: RNA Binding Motif Protein 15/15B;
ZCCHC4: Zinc Finger CCCH-Type Containing 4
ZC3H13: Zinc Finger CCCH-Type Containing 13
FTO: Fat mass and obesity-associated protein
ALKBH5: AlkB homolog 5
YTH: YT521-B homology
HNRNP: Heterogeneous nuclear ribonucleoprotein
IGF2BPs: Insulin-like growth factor 2 mRNA-binding proteins
EIF3: Eukaryotic translation initiation factor 3
PRRC2A: Proline rich coiled-coil 2A
SND1: Staphylococcal nuclease and tudor domain containing 1
RBPs: RNA binding proteins
carRNAs: Chromosome-associated regulatory RNAs
pri-miRNAs: primary miRNAs
ELAVL1: ELAV like RNA binding protein 1
MATR3: Matrin 3
PABPC1: Poly(A) binding protein cytoplasmic 1
UTR: Untranslated region
eIF3h: Eukaryotic translation initiation factor 3 subunit h
EcRNAs: Exonic circRNAs
EIcRNAs: Exon-intron circRNAs
ciRNAs: Intronic circRNAs
MeCP2: Methyl CpG binding protein 2
ceRNA: Competing endogenous RNA
IRES: Internal ribosome entry site
Ago2: Argonaute 2
RISC: RNA-induced silencing complex
